# Efficacy of computer- and/or internet-based cognitive-behavioral guided self-management for depression in adults: a systematic review and meta-analysis of randomized controlled trials

**DOI:** 10.1186/s12888-022-04325-z

**Published:** 2022-11-24

**Authors:** Megi Mamukashvili-Delau, Nicole Koburger, Sandra Dietrich, Christine Rummel-Kluge

**Affiliations:** 1 Department of Psychiatry and Psychotherapy, Klinik Und Poliklinik Für Psychiatrie Und Psychotherapie, Medical Faculty, Leipzig University, Semmelweisstraße 10, Haus 13, 04103 Leipzig, Leipzig, Germany; 2 Department of Psychiatry and Psychotherapy, Universitätsklinikum Leipzig, Leipzig, Germany; 3 Department of Personnel Development and Academic Personnel Development, Leipzig University, Leipzig, Germany; 4 Leipzig Travel, Leipzig Tourismus and Marketing GmbH, Leipzig, Germany

**Keywords:** Depression, CBT self-help, Internet/computer-based therapy, Minimal guidance

## Abstract

**Background:**

Depression is a worldwide disease. CBT-based self-help treatment allows patients with mild to moderate depression symptoms to improve their depression or to bridge the waiting- or pandemic period until they receive further clinical treatment.

**Objective:**

This systematic review and meta-analysis aims to explore the efficacy, acceptability and improvement in quality of life of computer-delivered and/or internet-based CBT self-help interventions with minimal guidance (up to 10 min) for depression. The second aim was to compare the effectiveness of reducing depression symptoms at post-treatment of treatment by the type of minimal guidance: (1) e-mail, (2) telephone calls, (3) e-mail and telephone together, or (4) face-to-face.

**Methods:**

The Cochrane depression, anxiety, and neurosis review group’s specialized register electronic searches, grey literature, reference lists and correspondence were used to search for published and unpublished RCTs that reported efficacy of computer- and/or internet-based CBT self-help treatments for depression with minimal guidance up to 10 min per week. Methodological quality of included studies was evaluated with Cochrane Collaboration tools for assessing risk of bias. The meta-analysis was accomplished using the RevMen software.

**Results:**

In total, 2809 study abstracts were checked for eligibility. Out of these, 19 studies (21 samples) with a total of 3226 participants were included. The results showed that concerning efficacy, the treatment group is superior to the control group with a medium to large effect size of 0.65. Also, treatment groups with combined guidance by e-mail and telephone calls together had greater effects (SMD -0.76) than groups with other types of minimal guidance (guided by e-mail SMD -0.63; guided face to-face SMD – 0.66; guided by telephone calls SMD -0.49). Findings showed also, that iCBT with minimal guidance had small but statistically significant effect size of 0.28 in improving quality of life. Moreover, there were higher drop-out rates in the treatment condition (RR 1.36) than in the control groups.

**Conclusions:**

The results of this meta-analysis support the efficacy of computer- and/or internet-based CBT self-help programs with minimal weekly guidance up to only 10 min for improving depression symptoms at post-treatment for adults.

In addition, the results are pointing towards two practical implications. Firstly, depressed persons can use self-help treatment with minimal guidance at home to improve their symptoms or to bridge the waiting time – or pandemic period – before they receive professional face-to-face treatment. Secondly, it can help clinicians to make the decision about using CBT-based self-help treatments for patients that do not need urgent professional treatment, or to combine it with face-to-face therapy.

## Introduction

Depression is a worldwide disease. It is associated with symptoms such as low mood, markedly diminished interest or pleasure in everything or almost everything, significant weight loss when not dieting or decrease in appetite nearly every day, a slowing down of thought and a reduction of physical movements, fatigue or loss of energy nearly every day, feelings of worthlessness or excessive or inappropriate guilt, diminished ability to think or concentrate, or indecisiveness, recurrent thoughts of death, recurrent suicidal ideation without a specific plan, or a suicide attempt or a specific plan for committing suicide, etc. [1].

All over the world, more than 264 million people of all ages are suffering from depression. Depression can lead to suicide, which is the second leading cause of death of adolescents globally [2]. Therefore, the risks of depression must be taken very seriously. Despite numerous studies in this field, there is still a need to explore more about depression and its treatment options to have sufficient tools for treating depression.

As the prognosis and evidence show, depression could become one of the largest determinants of disability in the world in the future. Pharmacotherapy is an effective treatment for depression disorders [3], but many patients do not want to take medication, or if they have taken medication, they might have experienced side effects or shown poor compliance. An effective alternative or addition to pharmacotherapy is psychotherapy, in particular cognitive-behavioral therapy (CBT) [4].

Despite the existence of effective evidence-based therapies, a large amount of depressed individuals (> 70%) don’t seek treatment for many reasons, such as perceived stigma, unavailability of clinicians or long waiting lists for the clinician treatment, probable prohibitive costs or geographic distance [5, 6].

A CBT-based self-help therapy can solve these eventual problems. Self-help treatments offer patients with depression brief and structured therapy with or without any contact with therapists. This kind of therapy can be received at home and among other things, it is relatively anonymous, it might help to avoid stigma and can be used according to the patient’s own schedules and needs.

Moreover, self-help treatments can help the patients to develop usable skills to identify and monitor problematic thoughts and emotions and to cope with them [7]. During the CBT-based self-help therapy, patients with mild to moderate depression symptoms can have a chance to reduce the severity of their depression symptoms or to bridge the waiting period or pandemic period until they receive the clinical (face-to-face) treatment.

Although there is a growing amount of studies with randomized controlled trials (RCTs) [4, 8–11] and meta-analyses [12–16] about evidence for the effectiveness of internet-based self-help, there is a lack of meta-analyses that aim to analyze the effectiveness of iCBT with minimal guidance. Although, there are some recent studies [17–19] reporting about reduced risk of depression symptoms deterioration in case of using internet-based guided self-help compared to control groups.

In addition, CBT-based self-help can be used as a stand-alone intervention as well as with different levels of support, which can be implemented in different forms, such as brief phone calls, postcards, short messages, or e-mails [20]. Minimal guidance by a mental health professional or by a psychotherapist can possibly increase the patients’ motivation to continue self-help and does not require a great deal of time. There are some studies in this field that report a higher efficacy of guided self-help interventions compared to unguided ones [20–24]. However, there is also a study, which reports an equal efficacy of supported and unsupported self-help programs for the interventions that included the program plus provider as treatment as usual (TAU) [25].

The decision to use self-help with minimal weekly guidance should be based on up-to-date, reliable, relevant and critical research. Hence, analyzing the efficacy of iCBT for depression with weekly minimal guidance of maximal 10 min per week may help clinicians (and not only) to make a decision about using this type of treatments if it is required. In addition to that, it would be also very important to analyses what kind of minimal guidance brings to more significant improvements in reducing of symptoms of depression.

Thus, the aim of this meta-analysis is to explore whether computer-delivered and/or web-based (i.e., internet-based) self-help interventions with minimal guidance of up to 10 min for adults and adolescents are effective in improving depression symptoms.

Moreover, we want to investigate which type of minimal guidance, (e.g. by e-mail, telephone calls, e-mails and telephone calls combined together, or face to-face minimal support) is more effective in reducing depression symptoms at post-treatment. Furthermore, we investigate the treatment acceptability as well as the change of quality of life.

## Methods

The methods of this meta-analysis refer to an original, broader Cochrane meta-analysis [26] and aim to summarize the depression outcomes of the studies that have put their focus on computer- and/or internet-based self-help programs for depression with weekly minimal guidance (up to 10 min) in adults and adolescence.

### Search methodology and identification trials

The following databases were searched: the Cochrane depression, anxiety, and neurosis review group’s specialized register (CCDANCTR). The CCDANCTR-References Register contains over 34,000 reports of RCTs in depression, anxiety and neurosis. Reports of trials for inclusion in the Group's registers are collated from routine (weekly), generic searches of MEDLINE (1950 -), EMBASE (1974 -) and PsycINFO (1967 -); quarterly searches of the Cochrane Central Register of Controlled Trials (CENTRAL), and review-specific searches of additional databases.

We searched the CCDANCTR-Studies Register using the following controlled vocabulary terms: CONDITION = (depressi* or dysthymi* or “mood disorder*” or “affective disorder*” or “affective symptoms”) AND INTERVENTION = (self* or bibliotherap* or computer* or web* or internet*) AND COMPARATOR = (treatment-as-usual or “usual care” or wait* or “attention control” or “attention placebo” or "minimal contact" or "minimal treatment" or (no NEAR2 intervention)). We searched the CCDANCTR-References Register using a more sensitive set of terms to find additional untagged/uncoded references: #1. (depress* or dysthymi* or mood* or “affective disorder*” or “affective symptoms” or mental or psychiatric):ti #2. ((depressi* NEAR2 (major or disorder)) or MDD):ab,kw,ky,mh,emt,mc,mh #3 (#1 or #2) #4. (self NEXT (care or chang* or direct* or guid* or unguid* or non- guid* or help or intervention or instruct* or manage* or *therap* or train* or treat*)):ab,kw,ky,mh,emt,mc #5. (audio* or bibliotherap* or book* or cCBT or iCBT or CD or CD-ROM or “chat room” or computer* or cyber* or DVD or e- mail or email or eHealth* or e-Health* or “electronic health” orinternet* or interactive or interapy or manual or manualised or "minim* guidance" or "minim* contact" or mobile or multimedia or multi-media or online or on-line or pamphlet or pamphlets or standalone or stand-alone or tape or taped or telemed* or telehealth* or "text messag*" or texting or “instant messag*” or video* or virtual or web* or www or “beat* the blues" or “blues away”):ti,ab,kw,ky,mh,emt,mc,mh #6. (#4 or #5) #7. (“treatment as usual” or "minim* contact*" or "minim* treatment*" or waitlist* or (wait* NEXT list*) or (placebo NEXT (attention or control or psyc* or *therap*)) or “attention control*” OR ((usual or non or "no" or delay*) NEAR2 (attention or *care or counsel* or intervention* or medicat* or support or treat* or *therap* or train*)) or untreat* or un- treat*):ti,ab,kw,ky,emt,mh,mc,mh or TAU.ab. #8. (#3 and #6 and #7) [Key—ti:title; ab:abstract; kw,ky:keyword fields; emt:EMTREE Headings; MH: Medical Subject Headings (MeSH); mc:MeSH checkwords.

We searched international trial registries via the WHO's trials portal (ICTRP) and ClinicalTrials.gov to identify unpublished or ongoing studies. We did not impose any restriction on date, language or publication status applied to the searches. Searching other resources like Grey literature. We searched sources of grey literature, including dissertations and theses, clinical guidelines and reports from regulatory agencies (where appropriate). ProQuest Dissertations and Theses Database, National Guideline Clearing House [27], Open Grey [28].

We checked the reference lists of all included studies and relevant systematic reviews to identify additional studies missed from the original electronic searches (for example, unpublished or in-press citations). We will also conduct a cited reference search on the Web of Science.

We contacted trialists and subject experts for information on unpublished or ongoing studies, or to request additional trial data. (For more information see [26]).

Studies met the following selection criteria:

#### Types of studies

Published or unpublished RCTs-, as well as crossover trials.

#### Types of participants

Participants from any ethnic groups aged 14 years or above with a clinically diagnosed depression, i.e. measured by standardized diagnostic criteria, or validated depression questionnaires, or both.

#### Types of interventions

Studies with experimental computer- and/or internet-based CBT self-help programs with weekly minimal guidance (i.e. up to 10 min) given by a mental health professional or a therapist. As control comparisons were eligible: treatment as usual, waiting list/delayed treatment condition, attention placebo, and psychological placebo.

#### Diagnosis

Studies with the following diagnostic criteria for depression were included: ICD-9 [29], ICD-10 [30], DSM-III [31], DSM-IV [32], and DSM-V [33]. The following questionnaires were accepted: Patient Health Questionnaire (PHQ) [34], Beck Depression Inventory (BDI) [35, 36], Hamilton Depression Rating Scale (HDRS) [37], and Montgomery Depression Scale (MADRS) [38], The Center for Epidemiologic Studies Depression Scale (CES-D) [39], Hospital Anxiety and Depression Scales (HADS) [40], Kessler Psychological Distress Scale (K-10) [41], Depression Anxiety Stress Scales (DASS) [42], or any other validated depression scale. If studies reported more than one type of depression outcome measure, those outcomes were extracted with the highest priority according to the following list: (1) PHQ-9; (2) BDI-II; (3) HDRS; (4) MADRS; (5) CES-D; (6) HADS.

#### Co-morbidities

Studies where the focus was on the depression diagnosis were eligible for inclusion.

#### Setting

Studies conducted in community, primary, secondary or tertiary services were all eligible for inclusion.

#### Types of outcome measures

##### Primary outcome

Treatment efficacy: changes in depressive symptomatology measured by validated depression scales.

##### Secondary outcomes

Comparison of the effectiveness of computer- and/or internet-based CBT self-help treatment by the type of minimal guidance: (1) by e-mail, (2) by telephone, (3) by e-mail and telephone together, or (4) face-to-face.

In addition, treatment acceptability – the number of participants who dropped out from the original study for any reason.

Furthermore, improvement in quality of life, as assessed with the use of validated measures.

### Data collection and analysis

The searching and analyzing process for this meta-analysis took place between 2015 and 2022. The search process resulted in 2606 study abstracts from CCDANCTR, another 203 studies from electronic searches, cross-referencing and grey literature. In total, 2809 study abstracts were checked for eligibility. A total o 2746 studies did not meet one or more of the inclusion criteria of this meta-analysis and were excluded. 41 studies could not be included or excluded because of a lack of required information described in the original studies or because there was no publication available to decide on inclusion or exclusion. These study authors were contacted a few times during the process of the meta-analysis. Either there was no response from them, or they could not provide sufficient information for making the decision to exclude or include these studies. Therefore, they are still in the awaiting assessment list (AA). There were also three ongoing studies found [43–45], that were in process at the time of conducting this meta-analysis.

**Fig. 1:**
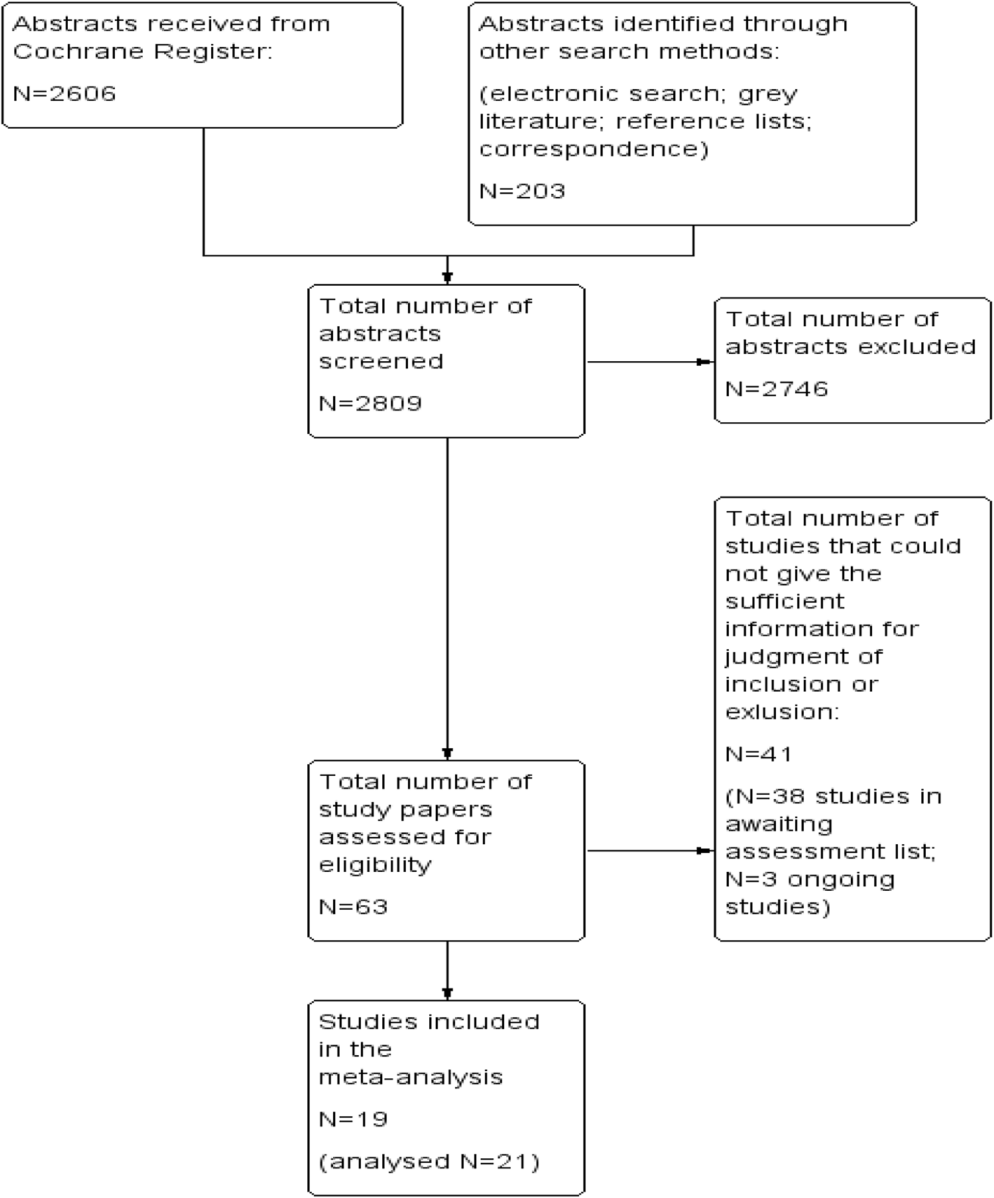
PRISMA flowchart outlining process of the meta-analysis

Finally, 19 studies [5, 8, 10, 46–61] met all criteria for inclusion. The results of two included study [46, 61] could be used as two separate samples due to its three-arm design. Therefore, in this meta-analysis a total of 18 samples were included. All studies included in this meta-analysis were RCTs. Figure 1 outlines the search process.

Data extraction was performed by using a data collection form/template based on the Cochrane Collaboration [62]. The information about characteristics of the included studies, such as means and standard deviation of the treatment efficacy for post intervention and the number of participants were extracted and entered into the Review Manager (RevMen), a software of Cochrane [63]. This program is used for preparing and maintaining reviews and meta-analyses. Included study authors were contacted to clarify exact information needed for this meta-analysis.

Besides, the study efficacy data were also differentiated by type of minimal guidance: (1) by e-mail, (2) by telephone calls, (3) by e-mail and telephone together, and (4) face-to-face.

The quality of the individual studies was assessed with the "Cochrane tool for assessing risk of bias" of the Cochrane Collaboration [64]. The studies were assessed using seven categories of risk of bias: random sequence generation, allocation concealment, blinding of participants and personnel, blinding of outcome assessment, incomplete outcome data, selective outcome reporting, and other bias. Each category was rated as low, high, or unclear.

### Statistical Analysis

Continuous data were analyzed as mean differences (MDs) or standardized mean differences (SMDs) and 95% confidence interval (CIs). The data were entered with a consistent direction of effect. Where studies had used the same outcome measure for comparison, data were pooled by calculating the MD. Where different measures were used to assess the same outcome, these data were pooled with SMDs and 95% CIs [64, 65].

We tested statistical heterogeneity between studies using a standard Chi^2^ test (with a significance level of alpha being less than or equal to 0.1 to indicate heterogeneity). We examined the I^2^ value using the following overlapping bands provided in the Cochrane Handbook for Systematic Reviews of Interventions [64, 65]: 0% to 40%: might not be important, 30% to 60%: may represent moderate heterogeneity, 50% to 90%: may represent substantial heterogeneity, 75% to 100%: may represent considerable heterogeneity.

### Data synthesis

The random-effect-model of meta-analysis was used as studies were estimating different treatment effects.

## Results

### Characteristics of included studies

The research yielded 19 studies (21 samples). All studies included in this meta-analysis were RCTs where participants were randomized into two or more groups (treatment vs. control wait list/ delayed treatment, TAU, etc. [26]). The number of participants that participated in the original studies was 3954. The data of 728 participants were not usable/eligible for our meta-analysis due to having no depression diagnosis at baseline or not receiving CBT-based self-help treatment with weekly minimal guidance. So, we analyzed the data of 3226 participants.

The participants of 15 studies [5, 8, 10, 46–52, 54, 56, 58, 60, 61] were recruited through the community. The participants of the further four studies were recruited through primary care [53], outpatients [57], community plus outpatients [59], and community plus primary care [55].

Studies included in this meta-analysis used measures of treatment efficacy—changes in depressive symptomatology measured by validated depression scales. The participants in every included sample received computer- and/or internet-based therapy with minimal guidance (up to 10 min per session/week). In six samples [8, 10, 47, 56, 57, 59] minimal guidance was received only by e-mails, participants of another seven samples (five studies) [5, 46, 48, 50, 61] were guided only by telephone calls, participants of six samples [49, 51, 52, 55, 58, 60] received combined guidance – by emails and by telephone calls together, participants of two samples [53, 54] were guided only face-to-face.

In these 21 samples, 15 different computer- and/or internet-based CBT self-help programs were used. Table**1**. provides a detailed overview of other characteristics of the included studies, such as number of participants included in original studies, depression severity of included participants, mean age with SDs and ethnicity of participants (so, as they were defined in original studies).

**Table 1:** Detailed characteristics of included studies

Study Author (Year)	N (Total number of participants)	Severity of depression	Mean Age (SD)	Intervention program	Control condition	Ethnicity
Andersson et al., 2005 [47]	117	Mild to moderate	Treatment group: m = 36.4(11.5) Control group: m = 36.3(9.9)	Internet-administered cognitive-behavioural self-help	Wait-list	Swiss
Berger 2011 [8]	76	Mild Moderate Severe Dysthymic disorder	Total sample: m = 38.8(14.0)	Internet-based self-help program (Deprexis)	Wait-list	Swiss German
Choi et al., 2012 [48]	63	Major depressive episode	Total sample: m = 39.0(11.07)	Internet-delivered CBT program (The Brighten Your Mood Program)	Wait-list	Resident of Australia, but self-identified as of Chinese origin
Clarcke et al., 2005 [49]	255	Major depression	Treatment group: m = 44.5(10.5) Control group: m = 45.0(10.6)	CBT-based ODIN program	Treatment as usual	Not defined
Farrer et al., 2011 [50]	155 (relevant for us *n* = 80)	Mild and higher	Treatment group: m = 41.7(12.1) Control group: m = 43.7(12.3)	Web-based CBT (Web with tracking)	Not active control condition	Not defined
Gilbody et al., 2015 [46]	691 (relevant for us *n* = 452)	Moderate	Total sample: m = 39.86(12.65)	(1) Beating the Blues (2) MoodGYM	Usual GP care	UK
Klein et al., 2016 [10]	1013	Mild to moderate	No means reported (participants were between 18 and 65 years of age)	Internet-program (Deprexis)	Care as usual alone	Not defined
Lambert et al. 2018 [60]	62	Moderate to severe	Treatment group: m = 39.3(12.0) Control group: m = 36.9(12.6)	Web-based course (eMotion)	Wait-list	UK
Mantani et al., 2017 [57]	164 (relevant for us *n* = 117)	Major	No means reported (participants were between 25 and 59 years of age)	Smartphone CBT program (Kokoro-App)	Switch alone arm (control arm)	Japanese
Mohr et al., 2013 [51]	102 (relevant for us *n* = 67)	Major depression	Treatment group: m = 47.6(12.4) Control group: m = 48.49(11.7)	MoodManager	Wait-list	African American; White; Other
Newby et al., 2013 [52]	109	Major	Total sample: m = 44.3(12.2)	Worrying and Sadness internet-program	Wait-list	Not defined
Newby et al., 2017 [58]	106	Moderate to severe	Total sample: m = 47.0(12.61)	Cartoon-style Web-based lessons teaching CBT skills	Treatment as usual	Not defined
Proudfoot et al., 2004 [53]	274	Mild Moderate Severe	Treatment group: m = 43.6(14.3) Control group: m = 43.4(13.7)	Beating the Blues	Treatment as usual	White; Bangladeshi; Black African; Black Caribbean; Black other; Indian; Pakistani; Other
Rosso et al., 2017 [5]	77	Major	Treatment group: m = 29.2(7.69) Control group: m = 28.8(6.74)	Internet-based sadness program	A monitored attention control group	Americans
Selmi et al., 1991 [54]	36 (relevant for us *n* = 24)	Major Mainor Intermittent	Total sample: m = 28.2(not reported)	Computer-administred treatment	Wait-list	All white
Smith et al., 2017 [59]	270 (relevant for us n = 129)	Major	Treatment group: m = 42.5(12.63) Control group: m = 37.59(13.29)	Internet-based sadness program	Wait-list	Not defined
Stiles-Shields et al. 2019 [61]	30	Moderate to severe	No means reported (participants were at least 18 years old)	(1) Boost Me (2) Thought Challenger	Wait-list	American
Titov et al., 2010 [55]	141 (relevant for us *n* = 94)	Major	Total sample: m = 43.0(12.86)	Internet-based sadness program	Delayed treatment group	Not defined
Van Straten et al., 2008 [56]	213 (relevant for us *n* = 81)	Major	No means reported (adults)	Web-based problem-solving program	Wait-list	Dutch
**Total:**	3954 (relevant for this meta-analysis *n* = 3226)	Mild to severe depression				

### Quality of included studies

**Fig. 2:**
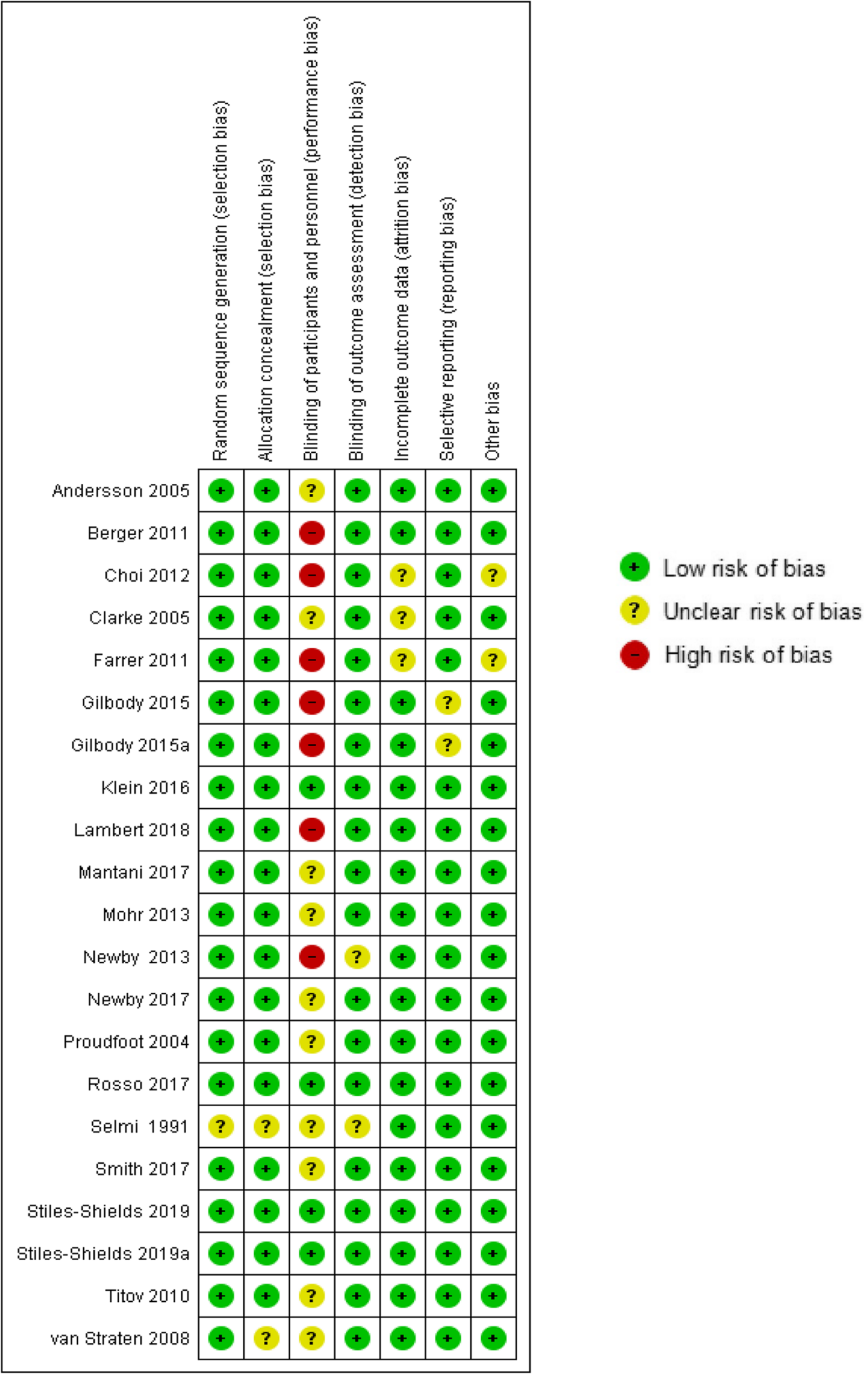
Summary of risk of bias identified for each included study

The risk of bias of all included studies could be assessed as ‘low to moderate’ due to the publications that had some unclear or high risk of bias (see Fig. 2). If there was no sign of bias, it was assessed as ‘low risk of bias’. Some of the biases in original studies were assessed as unclear because of insufficient information to judge existing bias, or it was assessed as ‘high risk of bias’ due to suspect of real existing bias.

The participants of all included studies were randomized and all studies except one [54] described their randomization method. The process of random allocation sequence was circumstantially described in all of the included studies except two [54, 56]. Therefore, there was a low risk of selection bias.

If participants as well as personnel were blinded, risk of performance bias was assessed as low. If personnel were not blinded, it was assessed as high risk. If there was not sufficient information about blinding of personnel, performance bias was assessed as unclear. In most of the included studies except three [5, 10, 61], this kind of bias was assessed as high or unclear.

There was a low risk of detection bias in most of the included studies except two [5, 54]. In these two studies, there was not sufficient information provided to permit judgement about blinding of outcome assessment.

Three studies [48–50] did not report sufficient information to judge about risk of attrition bias. The remaining studies had a low risk of incomplete outcome data.

All included studies except one [46] reported all predefined outcomes. Therefore, there was a low risk of reporting bias.

Finally, there was no sign of high risk of other sources of bias. Two studies [48, 50] did not report sufficient information about other bias.

### Test of heterogeneity

There are two ways to interpret the results of a meta-analysis: the random effect model and the fixed effect model. We chose to use the random effect model. In most real situations, the random effect model is more suitable as it assumes that effect sizes are estimates of their own “true” effect sizes, distributed around an average true effect, where variance is attributable to both sampling error and “real” between study variance. In contrast, the fixed effect model assumes that the effect sizes are all estimates of a single “true” effect size and that the variance between effect sizes is attributable to sampling error only. It is therefore more appropriate to use the random effect model.

For this reason, the heterogeneity of the effect size samples was automatically tested in RevMen with I^2^-values for every outcome.

**Fig. 3:**
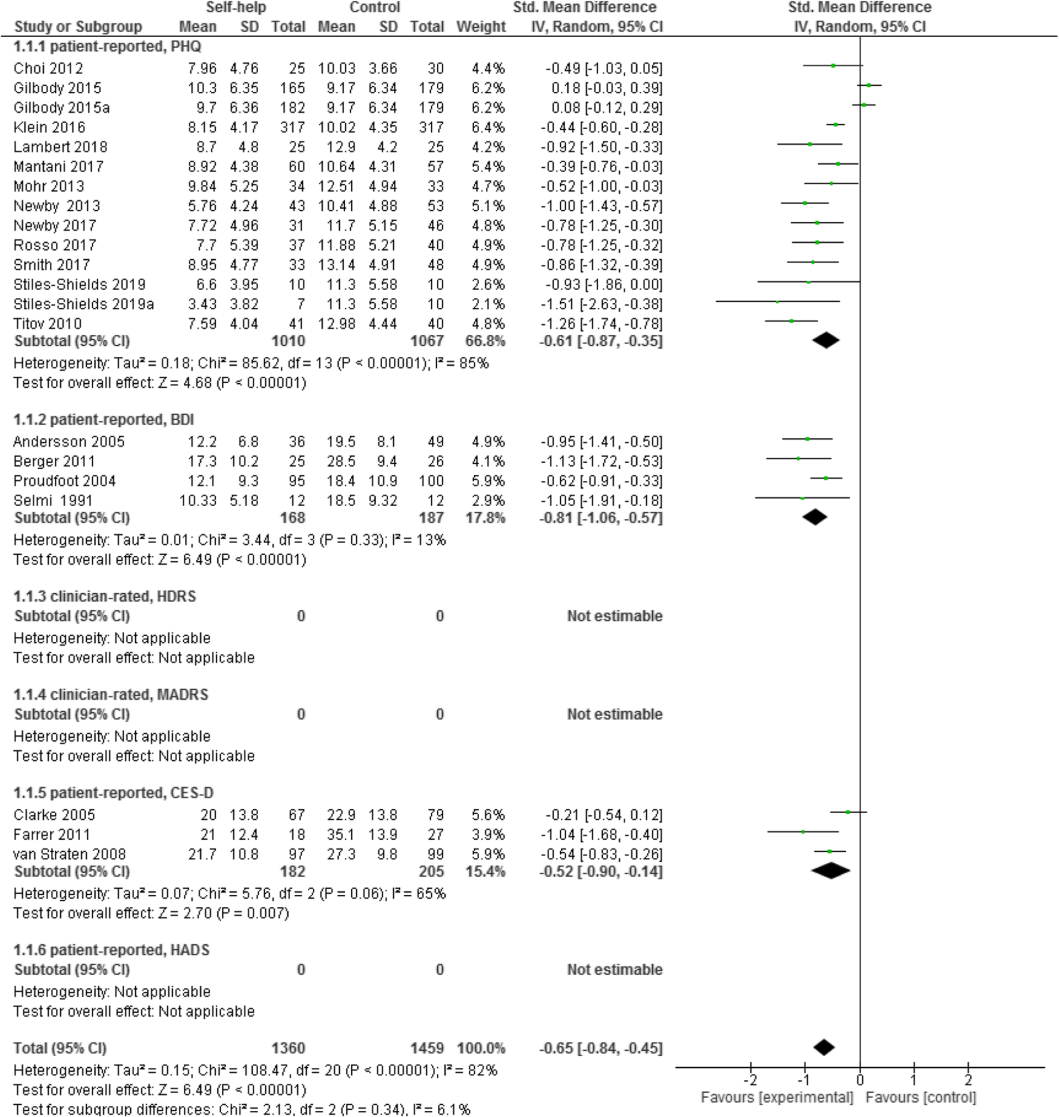
Forest plot of standardized mean difference (95% CI) in change of depressive symptoms for intervention and control conditions

The results of the heterogeneity test for treatment efficacy (Fig. 3) confirm that the random effect model is the proper approach for the interpretation of the results of this outcome (I^2^ = 82%, *P* < 0.00001).

### Primary outcome

#### Treatment efficacy

A total of 19 studies (21 samples) assessed the efficacy of computer and/or web-based CBT self-help treatment with weekly minimal guidance (up to 10 min) among 1360 participants by comparing the control conditions with a total of 1459 participants using post-intervention end point scores of depressive symptoms.

The results of treatment efficacy represent depressive symptoms, that were measured by various depression scales, including PHQ [34]; BDI-II [35], and CES-D [39]. So, they were undertaken using a SMD.

Two studies [47, 51] reported only standard error (SE) and no standard deviation (SD) for treatment- and control conditions. Therefore, we calculated SDs for these two studies. The calculation was conducted as outlined in the Cochrane handbook [62].

The total results of all scales together show statistically significant differences between computer- and/or internet-based CBT self-help treatment group and comparative interventions. Namely, the treatment group is favored over the control group with medium to large effect size of 0.65 (*n* = 2819, 21 RCTs, SMD -0.65, 95% CI -0.84 to -0.45, Z = 6.49, *P* < 0.001; I^2^ = 82%, *P* < 0.001; see Fig. 3).

Moreover, the analysis of depression outcomes, using post intervention end point scores for separate scales of depression, also showed statistically significant differences between intervention and control conditions. Namely, the intervention condition is favored significantly over the control condition on every single depression scale (see Fig. 3).

Depression data assessed with PHQ [34] showed that computer/internet-based CBT self-help interventions had a medium effect size of 0.61 (*n* = 2077, 14 RCTs, SMD -0.61, 95% CI -0.87 to -0.35, Z = 4.68, *P* < 0.001; I^2^ = 85%, *P* < 0.001). Data assessed with BDI-II [35] had a large effect size of 0.81 (*n* = 355, 4 RCTs, SMD -0.81, 95% CI -1.06 to -0.57, Z = 6.49, *P* < 0.001; I^2^ = 13%, *P* = 0.33), and data assessed with CES-D [39] showed a medium effect size of 0.52 (*n* = 387, 3 RCTs, SMD -0.52, 95% CI -0.90 to -0.14, Z = 2.70, *P* = 0.007; I^2^ = 65%, *P* = 0.06).

### Secondary outcomes

#### Computer/internet-based self-help intervention guided per e-mail compared to control condition

By comparing the interventions with minimal support and control groups using depression symptoms at post-intervention as the dependent variable, six studies [8, 10, 47, 56, 57, 59] assessed the efficacy of e-mail supported computer/internet-based CBT self-help programs among 1164 participants. The results were undertaken using SMD because of various depression scales used in this analysis of included studies.

**Fig. 4:**
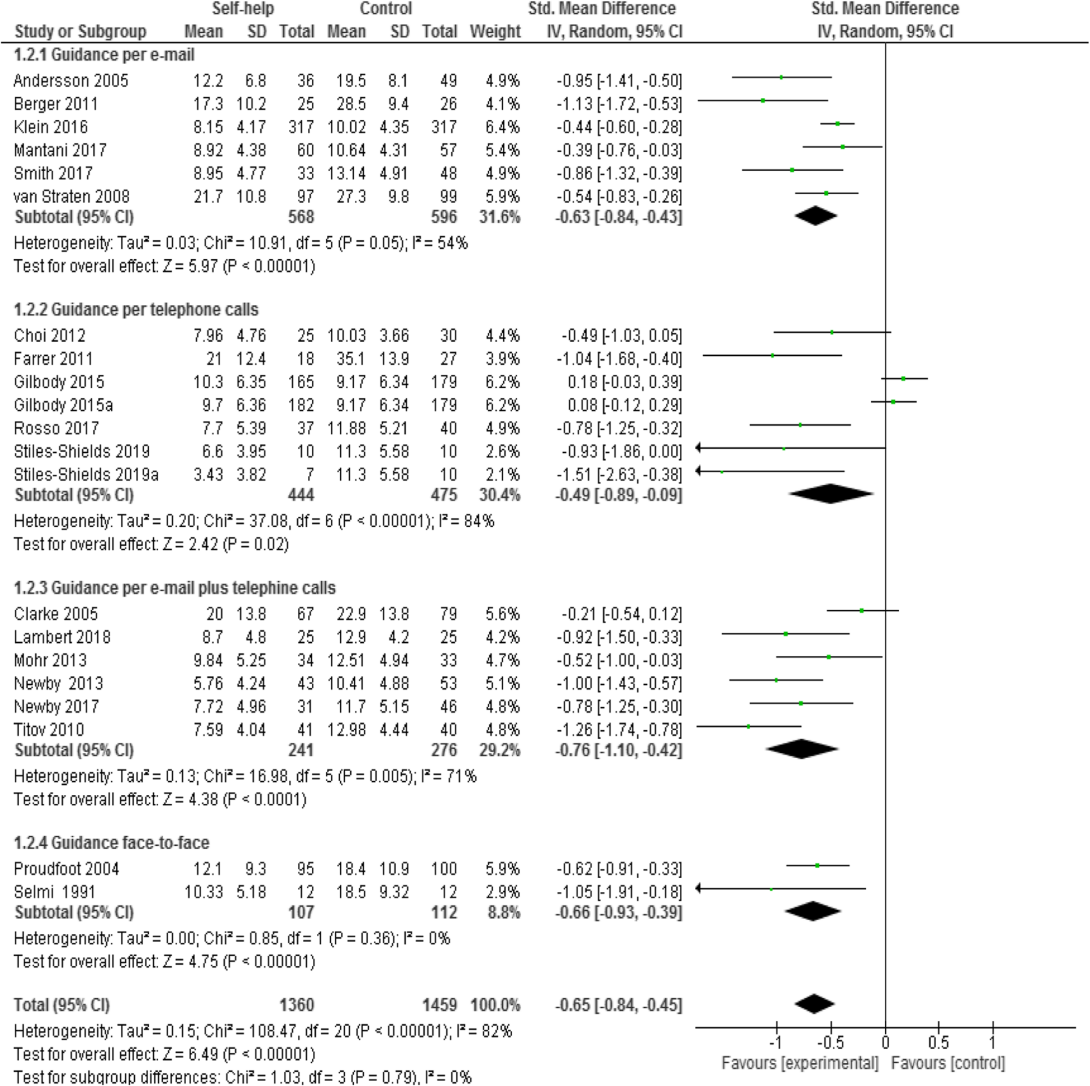
Forest plot of standardized mean difference (95% CI) in change of depression symptoms for self-help interventions with minimal guidance by e-mail, by telephone calls, by e-mail plus telephone calls and face-to-face and control groups

The result of this outcome showed a statistically significant difference between computer- and/or internet-based CBT self-help treatment groups with minimal guidance by e-mail and comparative interventions. The depression data of participants at post-treatment, guided by weekly e-mail contact, was favored over the control group with a medium to large effect size of 0.63 (*n* = 1164, 6 RCTs, SMD -0.63, 95% CI -0.84 to -0.43, Z = 5.97, *P* < 0.001; I^2^ = 54%, *P* = 0.05; see Fig. 4).

#### Computer/internet-based self-help intervention guided per telephone calls compared to control condition

A total of five studies (total seven samples) [5, 46, 48, 50, 61] assessed efficacy of computer/internet-based CBT self-help programs guided by telephone calls among 919 participants by comparing the intervention and control groups using depressive symptoms at post-intervention. The results were undertaken using SMD because of various depression scales used in this analysis of included studies.

The analysis showed that the participants in the intervention group, who received self-help intervention with minimal guidance per weekly phone calls, were favored over the control group; and the results were statistically significant (*n* = 919, 7 RCTs, SMD -0.49, 95% CI -0.89 to -0.09, Z = 2.42, *P* = 0.02; I^2^ = 84%, *P* < 0.00001; see Fig. 4).

#### Computer/internet-based self-help intervention guided per e-mail and telephone calls together compared to control condition

There were six studies [49, 51, 52, 55, 58, 60] that assessed the treatment efficacy of combined support—by e-mails and telephone calls together—of computer and/or internet-based CBT self-help interventions among 467 participants with depressive symptoms at post-intervention. The results were undertaken using SMD because of various depression scales used in this analysis of included studies.

The meta-analysis of these five studies showed a statistically significant difference between computer/internet-based CBT self-help treatment groups with minimal combined guidance and comparative interventions. Namely, treatment condition was favored over the control condition with medium to large effect size of 0.76 (*n* = 517, 7 RCTs, SMD -0.76, 95% CI -1.10 to -0.42, Z = 4.38, *P* < 0.0001; I^2^ = 71%, *P* = 0.005; see Fig. 4).

#### Computer/internet-based self-help intervention guided face-to-face compared to control condition

Only two studies [53, 54] assessed efficacy of face-to-face guided computer/internet-based CBT self-help programs among 219 participants with depression by comparing the intervention and control groups using depressive symptoms at post-intervention. The results were undertaken using SMD because of various depression scales used in this analysis of included studies.

The results showed a statistically significant difference between computer- and/or internet-based CBT self-help treatment groups with minimal face-to-face guidance and comparative interventions. The intervention group favored over the control group with a medium to large effect size of 0.66 (*n* = 219, 2 RCTs, SMD -0.66, 95% CI -0.93. to -0.39, Z = 4.75, *P* < 0.001; I^2^ = 0%, *P* = 0.36; see Fig. 4).

Meta-analysis on depression symptoms for these studies showed no heterogeneity (I^2^ = 0%).

#### Acceptability of computer/internet-based CBT self-help treatment with minimal guidance

Sixteen Studies (18 samples) [5, 8, 10, 46–48, 50, 52–55, 57–61] with a total 2879 participants assessed the outcome of treatment acceptability – the number of participants who dropped out from the original study for any reason.

**Fig. 5:**
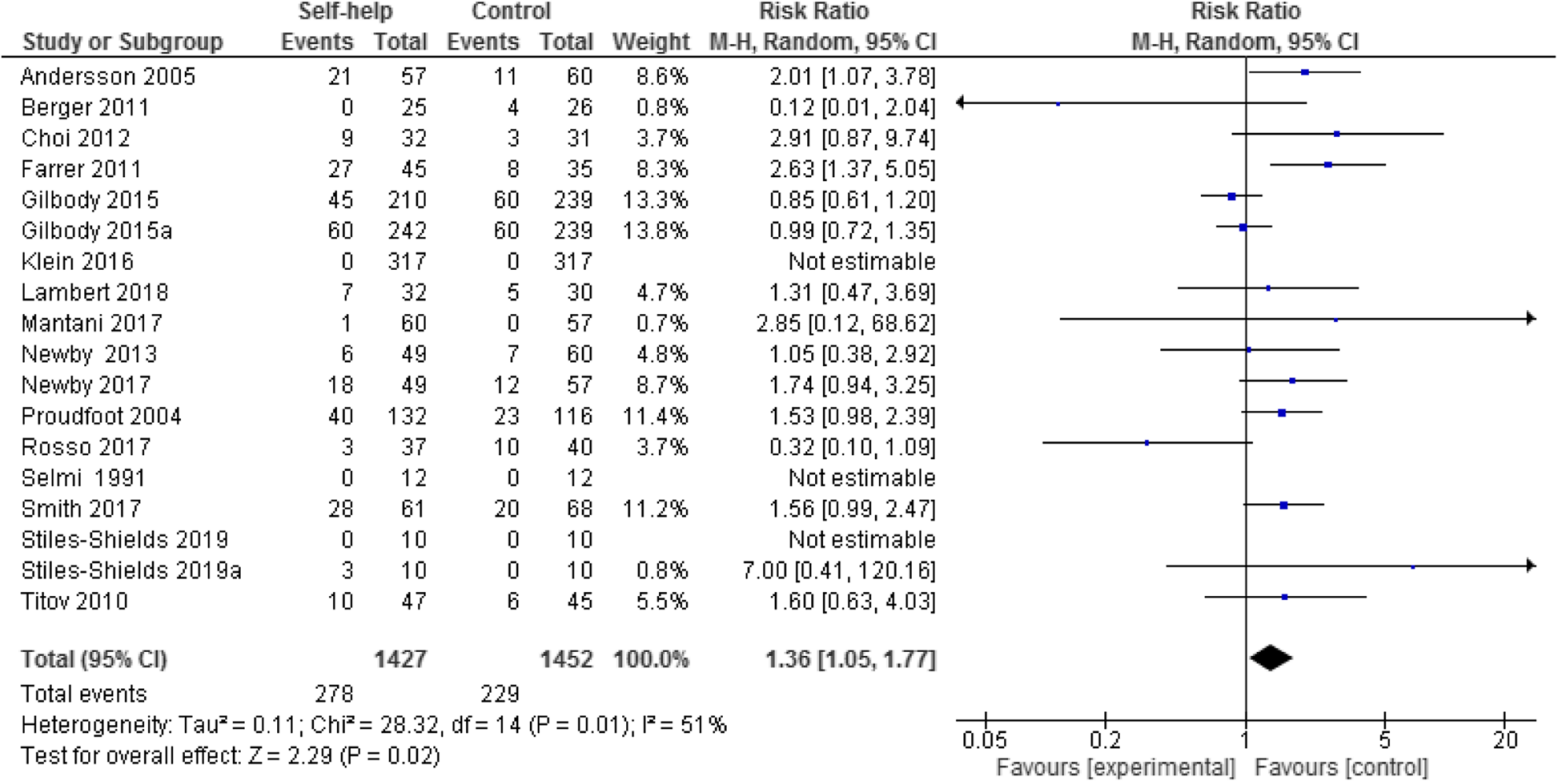
Forest plot of treatment acceptability: the number of participants who dropped out for any reason (Risk Ratio, 95%Cl)

The following forest plot (see Fig. 5) shows the relative chance of participants dropping out under treatment and control conditions.

Tree studies [49, 51, 56] did not report any information about the number of dropped out participants from baseline to post treatment. Therefore, it was not possible to include these three studies in this analysis. Since three studies [10, 54, 61] reported that there were no participants that dropped out from treatment or control groups, the data of these studies were not estimable for acceptability analysis.

We converted pooled odd ratios (ORs) to risk ratios (RRs), where the reported “events” represent the total number of dropped out participants in treatment- and control groups. Also, the total number of participants randomized in the treatment- and control groups is reported.

The results showed that the participants in the treatment condition were 1.36 times more likely to drop-out from the intervention condition than the participants in control condition (*n* = 2879, 18 RCTs, RR 1.36, CI 1.05 to 1.77, Z = 2.29, *P* = 0.02; I^2^ = 51%, *P* = 0.01, see Fig. 5). A total of 276 participants (19.34%) dropped out from the intervention condition early, compared to 229 participants (15.77%) in the control condition.

#### Improvement in quality of life

A total of 7 studies assessed the improvement of quality of life among 998 participants by comparing the control conditions with a total of 1031 participants using post-intervention end point scores (where poor scores mean low improvement in quality of life).

**Fig. 6:**
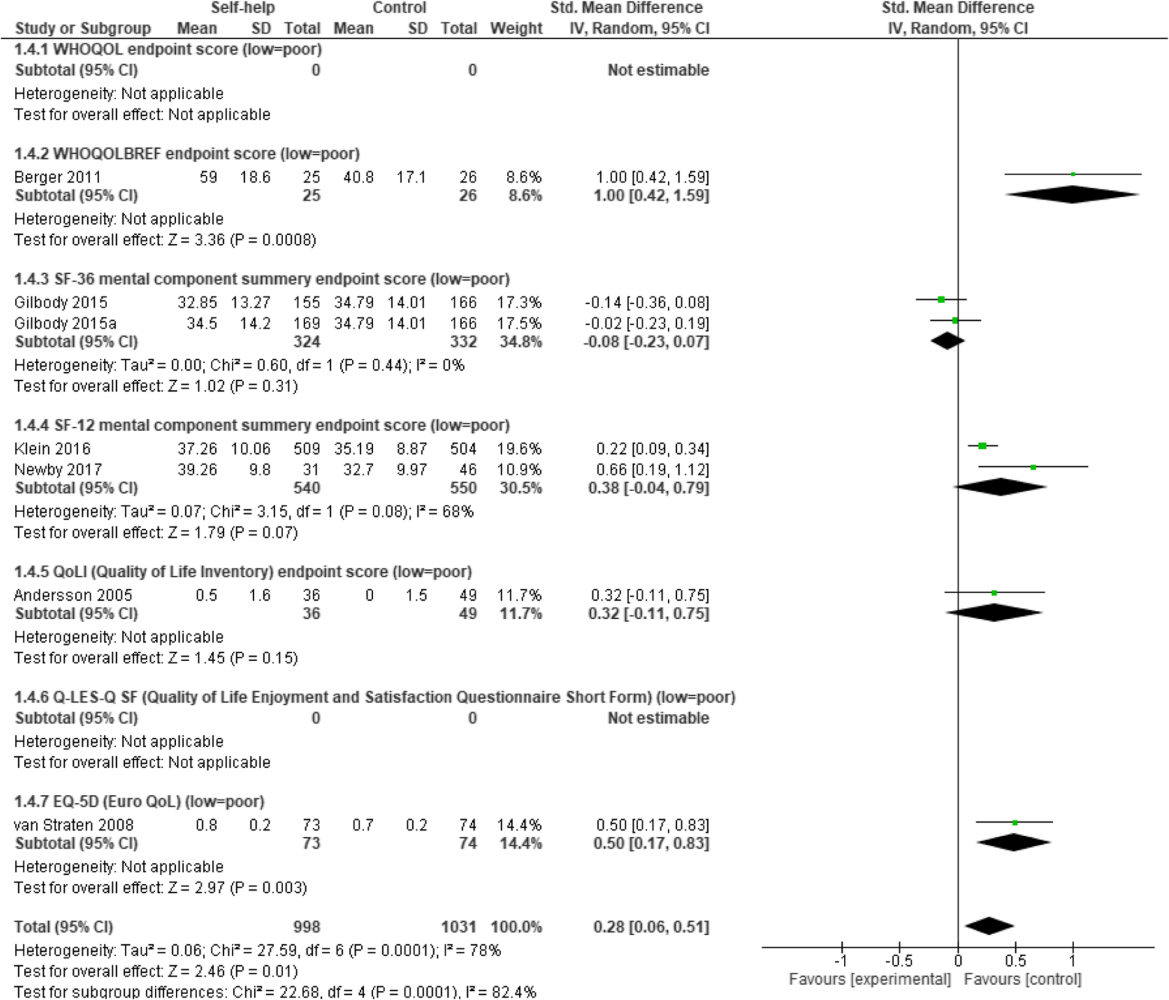
Forest plot of standardized mean difference (95% CI) in quality of life (low = poor) for intervention and control conditions

The total results of all scales together show statistically significant differences between computer- and/or internet-based CBT self-help treatment group and comparative interventions. Namely, the treatment group is favored over the control group with a low effect size of 0.28 (*n* = 2029, 7 RCTs, SMD 0.28, 95% CI 0.06 to 0.51, Z = 2.46, *P* = 0.01; I^2^ = 78%, *P* < 0.001; see Fig. 6).

## Discussion

### Efficacy of computer- and/or internet-based CBT self-help treatment with minimal guidance

In this meta-analysis we analyzed the efficacy of computer- and/or internet-based CBT self-help programs for depression with minimal guidance through 21 samples (19 RCTs) with a total of 3226 participants. The results revealed that the participants in the intervention group, who participated in CBT self-help programs with weekly minimal guidance, significantly improved their depression symptoms with medium to large effect size of -0.65 at post-intervention compared with those in the control group. This result approximates the effect size of 0.64 [66] and effect size of 0.63 [67] reported in previous meta-analyses. In a recent systematic review and meta-analysis [12] from 2021, it was also found that guided internet-based CBT was associated with more effectiveness in reducing depressive symptoms than control conditions.

Moreover, analyses of depression outcomes, using post-intervention end-point scores for individual scales of depression, also showed that the intervention group was favored significantly (PHQ [34] _ SMD -0.61; BDI-II [35] _ SMD -0.81; CES-D [39] _ SMD -0.52) over the control group.

In addition, the results of this analysis indicate a high degree of heterogeneity (I^2^ = 82%).

Hence, computer- and/or internet-based CBT self-help for depression with minimal weekly guidance (up to 10 min) can be useful in reducing depression symptoms for adults and adolescents. Firstly, this information can help patients with depression to receive a suitable self-help treatment and to bridge the waiting time for professional face-to-face treatment. Secondly, it would help the clinicians to make the decision about using CBT-based self-help treatments for patients who do not need urgent professional treatment, or to combine it with face-to-face therapy [11].

### Comparison of the effectiveness of computer- and/or internet-based self-help treatment for depression by the type of minimal guidance

A total of 19 Studies (21 samples) with 2819 participants were included for this analysis. Six studies assessed the efficacy of e-mail-supported computer/internet-based CBT self-help programs among 1164 participants. Five studies (total seven samples with 919 participants) assessed the efficacy of depression treatment supported by telephone calls. 517 participants in five studies were guided by e-mails and telephone calls together. 219 participants in two studies received face-to-face minimal guidance.

The findings of this analysis revealed that CBT-based self-help treatments for depression, provided by computer or internet, can be beneficial in reducing depression symptoms by every single type of guidance (described above). Although the participants in the treatment condition, who received weekly minimal guidance by e-mail (SMD -0.63), by e-mail and telephone calls together (SMD -0.76), face-to-face (SMD -0.66), or by telephone calls (SMD -0.49); showed significant improvements, with medium to large effect sizes in reducing their depression symptoms compared to participants in the control condition. Furthermore, the intervention group with a combination of guidance types, e.g. e-mails and telephone calls together, showed more statistically significant reduction of depression symptoms than any other treatment group compared to control group.

There are some studies [20, 50, 68, 69] or meta-analyses [12, 70, 71] in this field studying the necessity of guidance for more effectiveness of CBT-based self-help treatments for depression.

To our knowledge, no previous meta-analysis on self-help treatments for depression has compared the potential differences of the treatment groups, whilst distinguishing types of minimal guidance, e.g. by e-mail, by telephone calls, by e-mail and telephone calls together, or face-to-face minimal support. We could find only one study [72] that compared the effectiveness of internet-based CBT self-help guided either by telephone calls or e-mail correspondence (approx. 15 min per participant and week) among patients with major depression at post-treatment. In this study, no difference between these two groups was found. However, it should be noted that the previous study had a small sample size, limiting the statistical power to detect between-group differences.

Therefore, our findings may be very important for planning and making decisions about the support type of future computer- and/or internet-based CBT self-help interventions for depression.

### Acceptability of computer- and/or internet-based CBT self-help with minimal guidance

Participant drop-outs were reported in 16 studies (18 samples). The analysis of treatment acceptability showed that the participants in the treatment condition were 1.36 times more likely to drop out from the intervention condition than the participants in the control condition. In total, 19.34% of participants dropped out from the intervention and 15.77% from the control condition. These findings are consistent with the majority of studies or meta-analyses examining internet-based CBT self-help treatments [15, 18, 73–75].

Greater drop-outs in the treatment condition compared to the control condition could be caused by many factors: first, the self-help treatment with minimal guidance may have helped patients to reduce their depression severity before post-treatment measurements and they therefore had no need to continue the treatment. Second, self-help treatment required too much time and energy, or they had technical difficulties, e.g. in using the computer/internet. As far as the acceptability in the control group is concerned, one of the reasons why patients were less likely to drop out from the study compared to the treatment condition may be the promise of receiving adequate treatment after the waiting time (wait list control condition).

The drop-out rate for computer- and/or internet-based treatment condition found in this meta-analysis (19.34%) or in another meta-analysis (22%) [15] was even lower than the drop-out rate (24.63%) of a meta-analysis, which explored the effectiveness and drop-out rate of face-to-face CBT in outpatients [76].

### Improvement in quality of life

The analysis of seven RCTs with 2029 participants showed that iCBT with minimal guidance up to ten minutes had a small but statistically significant effect size of 0.28 in improving quality of life at post treatment compared to control conditions.

The findings of this outcome are well supported by a previous meta-analysis [14] that reported almost the same effect size of 0.29 of internet-based behavioural activation at the immediate posttest compared to control groups. Another meta-analysis [16] reported a medium (controlled) effect size (g = 0.56) of iCBT compared to the control condition.

### Strengths, limitations and implications for future search

In our reality with technologies, becoming more and more important in every part of our lives computer-based programs for mental health, and not only in this field, are gaining more and more relevance. Working from home or arranging everyday tasks from home became very popular and actual. So, computer-based self-help programs for people who are suffering from depression symptoms might be a smart healthcare offer to reduce these symptoms, or to prevent an increase in the severity of depression.

In order to minimize bias during the literature search and selection of publications, a clearly defined set of inclusion and exclusion criteria was used. The current review had clearly defined criteria regarding participants, intervention, study design, and outcomes.

Funnel plots were inspected for the outcomes measures to assess the likely presence of publication bias. There was no evidence of possible funnel plot asymmetry for either outcome. The graphs appeared to be symmetrical.

A low to moderate risk of bias was due to insufficient details reported in included studies. Moderate to high risk of bias was detected only in case of performance bias. However, it is very difficult or sometimes even impossible to achieve total blinding of personnel and participants in such psychotherapeutic studies.

This meta-analysis has several limitations that also may present an opportunity for areas of future research and practice. The most compelling limitations of the present meta-analysis are the limitations of the individual studies included.

Firstly, the included RCTs had been assessed as moderate to high in methodologic quality, which allows to conclude that the present meta-analysis is relatively free from critical bias. But, the risk of bias classification as high, low or unclear may have led to over- or underestimation in the results. The assessment of any risk of bias as ‘unclear risk of bias’, in reality, may have included potential of ‘high risk of bias’ or ‘low risk of bias’. A lack of detailed information described in some original studies about selection, performance, detection, attrition and outcome process could make it difficult to be assessed as a real risk of bias.

Secondly, although there were significant positive effects of self-help treatments in reducing depression symptoms, there were high drop-out rates reported in the original studies. Given that the number of dropped out participants from the study was higher in the treatment group than in the control condition, it would be very important for future research to examine the reasons provoking high drop-out rates in treatment as well as in control conditions.

Thirdly, different programs with different number of sessions of computer- and/or internet-based CBT self-help tend to report different effect sizes.

Fourthly, we evaluated the efficacy of CBT-based self-help programs only at post-treatment, i.e. only short-term benefits of computer- and/or internet-based CBT self-help programs with minimal guidance were investigated. The long-term benefits of these kind of programs remain unclear. For future research it would be very important to explore not only short-term but also long-term benefits.

In addition, our findings may be at risk of availability bias due to 41 studies, that are either ongoing or still awaiting assessment due to insufficient information for their inclusion or exclusion. Therefore, the possibility of missing data due to this insufficient information may limit our results.

Finally, the current meta-analysis included only published studies, out of which may arise the chance for publication bias. The potential for studies reporting small or null findings and not being published through either the reluctance from authors or journal editors dismissing them may be a problem. Publication bias is, however, a problem for all researchers and not only for this meta-analysis.

## Conclusions

The results of this meta-analysis support the efficacy of computer- and/or internet-based CBT self-help programs with minimal guidance of up to ten minutes for improving depression symptoms at post-treatment with medium to large effect. The comparison of effectiveness of these CBT-based self-help programs by type of weekly minimal guidance shows greater effects in reducing depression symptoms using combined guidance by e-mail and telephone calls together than using of other type of minimal guidance. However, the groups guided weekly by e-mail, by telephone calls or face-to-face also had significant improvements in reducing depression symptoms with medium to large effects. Furthermore, our findings showed, that iCBT with minimal guidance had a small but statistically significant effect size in improving quality of life. In addition, there were higher drop-out rates in the treatment condition than in the control groups. Future research should evaluate long-term benefits of computer- and/or internet-based CBT self-help programs with weekly minimal guidance for depression and the possible reasons for high drop-out rates in treatment as well as in control conditions. 

## Data Availability

The datasets used and/or analysed during the current review and meta-analysis available from the corresponding author on reasonable request.

## Abbreviations

RCTs: Randomized controlled trials
TAU: Treatment as usual
CCDANCTR: The Cochrane Depression, Anxiety, and Neurosis Review Group’s Specialised Register
ICD: International Statistical Classification of Diseases and Related Health Problems
DSM: Diagnostic and Statistical Manual of Mental Disorders
PHQ: Patient Health Questionnaire
BDI: Beck Depression Inventory
HDRS: Hamilton Depression Rating Scale
CES-D: The Center for Epidemiologic Studies Depression Scale
MADRS: Montgomery Depression Scale
K-10: Kessler Psychological Distress Scale
HADS: Hospital Anxiety and Depression Scales
DASS: Depression Anxiety Stress Scales
RevMen: Review Manager
MD: Mean Difference
SD: Standard Deviation
SE: Standard Error
SMD: Standardized Mean Difference
CI: Confidence Interval
OR: Odd Ratio
RR: Risk Ratio
